# In-Cell NMR of Intact Mammalian Cells Preserved with the Cryoprotectants DMSO and Glycerol Have Similar DNP Performance

**DOI:** 10.3389/fmolb.2021.789478

**Published:** 2022-01-25

**Authors:** Yiling Xiao, Rupam Ghosh, Kendra K. Frederick

**Affiliations:** ^1^ Department of Biophysics, UT Southwestern Medical Center, Dallas, TX, United States; ^2^ Center for Alzheimer’s and Neurodegenerative Disease, UT Southwestern Medical Center, Dallas, TX, United States

**Keywords:** Dynamic nuclear polarization (DNP), AMUPol, cryopreservation, DMSO (dimethyl sulphoxide), glycerol, in-cell NMR, HEK293

## Abstract

NMR has the resolution and specificity to determine atomic-level protein structures of isotopically-labeled proteins in complex environments and, with the sensitivity gains conferred by dynamic nuclear polarization (DNP), NMR has the sensitivity to detect proteins at their endogenous concentrations. Prior work established that DNP MAS NMR is compatible with cellular viability. However, in that work, 15% glycerol, rather than the more commonly used 10% DMSO, was used as the cellular cryoprotectant. Moreover, incubation of cells cryoprotected 15% glycerol with the polarization agent, AMUPol, resulted in an inhomogeneous distribution of AMUPol through the cellular biomass, which resulted in a spatial bias of the NMR peak intensities. Because 10% DMSO is not only the most used cryoprotectant for mammalian cells, but also because DMSO is often used to improve delivery of molecules to cells, we sought to characterize the DNP performance of cells that were incubated with AMUPol and cryoprotected with 10% DMSO. We found that, like cells preserved with 15% glycerol, cells preserved with 10% DMSO retain high viability during DNP MAS NMR experiments if they are frozen at a controlled rate. However, DMSO did not improve the dispersion of AMUPol throughout the cellular biomass. Cells preserved with 15% glycerol and with 10% DMSO had similar DNP performance for both the maximal DNP enhancements as well as the inhomogeneous dispersion of AMUPol throughout the cellular biomass. Therefore, 10% DMSO and 15% glycerol are both appropriate cryoprotectant systems for DNP-assisted MAS NMR of intact viable mammalian cells.

## Introduction

In-cell structural biology enables the study of protein conformation in environments that maintain the identity, stoichiometry, concentrations and organization of the myriad of biomolecules that can interact with a protein of interest. (Frederick et al., 2015; Theillet et al., 2016; Burmann et al., 2020; Luchinat et al., 2020) Capturing the effect of these complicated environments on biomolecular conformation is of particular importance for proteins that have more than one stable conformation, interact with cellular components or contain regions of intrinsic disorder. Nuclear Magnetic Resonance (NMR) is uniquely suited to study proteins in these complicated contexts with atomic level resolution. NMR spectroscopy detects only NMR-active nuclei. These nuclei are non-perturbative probes that can be specifically incorporated into a protein of interest that is either delivered to or expressed inside the cell. (Selenko et al., 2006; Inomata et al., 2009; Theillet et al., 2013; Majumder et al., 2015; Burmann et al., 2020) NMR has the resolution and specificity to determine atomic-level protein structures of isotopically-labeled proteins in complex environments (Sakakibara et al., 2009) and, with the sensitivity gains conferred by dynamic nuclear polarization (DNP), NMR has the sensitivity to detect proteins at their endogenous concentrations (Renault et al., 2012; Frederick et al., 2015; Albert et al., 2018; Costello et al., 2019; Narasimhan et al., 2019; Schlagnitweit et al., 2019).

We recently established that sample conditions that favor efficient DNP enhancements are compatible with cellular viability. In that work, we established methods that maintained cellular viability throughout the DNP NMR experiments and found that the magnitude of the sensitivity enhancements for such samples were high enough to enable detection of a protein at micromolar concentrations inside intact cells in experimentally tractable experimental times. (Ghosh et al., 2021) Briefly, cells were cryoprotected, transferred to rotors and frozen at the controlled rate of 1°C per minute before cryogenic transfer to the pre-cooled NMR spectrometer for analysis. (Ghosh et al., 2020; Ghosh et al., 2021) After structural characterization via DNP MAS NMR, these cells can be cultured or imaged and their phenotype can be determined and compared with cells before structural characterization. (Ghosh et al., 2021) However, that work only examined cells that were cryopreserved using 15% glycerol as the cryoprotectant. While the overall approach to sample preparation is likely to be generalizable to freezing media with different compositions, this has not been explicitly demonstrated. The most common cryoprotectant for cultured mammalian cells is dimethylsulfoxide (DMSO) at a concentration of 10% (v/v). Indeed, the handful of studies that examine preparations of mammalian cells using DNP NMR use DMSO (Albert et al., 2018; Narasimhan et al., 2019; Schlagnitweit et al., 2019; Overall et al., 2020), although the sample composition—including the choice of cryoprotectant—and post-experiment cellular viability, have only very recently been considered (Ghosh et al., 2020; Ghosh et al., 2021; Overall and Barnes, 2021). Given the widespread preference for 10% DMSO over 15% glycerol as the cryoprotectant for cellular cryopreservation, we sought to determine if cryoprotection using 10% DMSO could also support cellular viability throughout the DNP NMR experiments.

DNP increases the sensitivity of NMR spectroscopy through the transfer of the large spin polarization of an unpaired electron to nearby nuclei (Ni et al., 2013) which are typically introduced into a sample by doping with millimolar concentrations of stable biological radicals (Sauvée et al., 2013; Lund et al., 2020; Stevanato et al., 2020). The sensitivity enhancements from DNP rely upon proximity to a polarization agent. Thus, DNP-enhanced MAS NMR experiments are biased towards observation of molecules that are accessible to polarization agents. Despite how critical the dispersion of polarization agents in a sample is to both achieve high sensitivity and interpret the results, the dispersion of polarization agents in intact cells has only very recently been considered (Ghosh et al., 2021). In our recent work that described methods for DNP MAS NMR on viable cells we described two of many potential approaches to deliver polarization agents to intact cells. In that work, we introduced AMUPol to cells by incubation of intact cells with AMUPol and by electroporation of intact cells in the presence of AMUPol to transiently permeabilize the membrane. (Ghosh et al., 2021) We compared the distribution of AMUPol throughout the cellular biomass for cells prepared in these two different ways to the distribution of AMUPol throughout the cellular biomass for cellular lysates where the cellular membrane does not present a barrier to distribution. We found that while AMUPol was homogenously distributed in cellular lysates and cells where AMUPol had been introduced by electroporation. AMUPol was inhomogeneously distributed in cells where AMUPol was delivered by incubation. In samples of cells incubated with AMUPol, the signal intensity from DNA in the nucleus was lower than the signal intensity from proteins and RNA in the cytoplasm. Thus, data from experiments on such samples will report qualitatively, and not quantitatively, on the structural ensemble; any observed conformation in such samples certainly exists, but the relative population of that conformation to any other cannot be inferred from peak intensities. The method used to introduce the polarization agent affects the experimental result and therefore must be chosen to address the structural question under consideration. Interestingly, DMSO is not only often used as a cryoprotectant (Lovelock and Bishop, 1959) but is often also used as a cellular penetration enhancer (Williams and Barry, 2004). Here we assessed the performance of 10% DMSO to determine not only if it is able to support cellular viability throughout DNP MAS NMR but also to determine if it can improve delivery of the polarization agent, AMUPol, to the cell.

## Materials and Methods

### Sample Preparation

To reduce experimental acquisition times, we uniformly isotopically labeled HEK293 cells by culturing them in isotopically-enriched media. Human embryonic kidney 293 (HEK293) cells were cultured in ^13^C, ^15^N labelled media (BioExpress 6000 Mammalian U-^13^C, 98%; U-^15^N, 98%, Cambridge Isotope Laboratories, MA, United States) with 10% (v/v) fetal bovine serum (FBS, qualified, Gibco) and 1% (v/v) PenStrep (Gibco) at 37 °C and 5% CO_2._ Confluent plates were harvested using Tryp-LE Express (Gibco) and BioExpress 6000 media, transferred to 15 ml conical tube and centrifuged at 233 x *g* for 5 min at 22 °C using a swinging bucket rotor (Beckman Coulter). Pelleted cells were resuspended and washed once with 1x PBS (-CaCl_2_, -MgCl_2_, pH 7.4, Gibco). AMUPol was delivered to cells by incubation, to do so, a 50 μL cell pellet was mixed with 50 µL perdeuterated 1x PBS (85% D_2_O + 15% H_2_O, pH 7.4) containing AMUPol (Cortecnet, NY, United States) and 11 µL of *d*_*6*_-DMSO. The 111 μL cell suspension had a final composition of 10% (v/v) *d*_*6*_-DMSO, 76.5% (v/v) D_2_O and 13.5% (v/v) H_2_O. After delivery of AMUPol, cells were transferred into 3.2 mm sapphire rotor (Bruker) by centrifugation in a swinging bucket rotor at 100 x *g* for 2 min at 22 °C. The supernatant was removed, and rotors were frozen at a controlled rate (1 °C/min) in “Cool Cell LX” (Corning) in the –80 °C freezer for 12–16 h. Finally, frozen rotors were transferred to liquid nitrogen storage until measurement by DNP NMR.

### Trypan Blue Exclusion Assay

Pelleted cells (10 µL) were diluted into 100 µL unlabeled DMEM and 10 µL of this cell suspension were mixed with 10 µL of Trypan Blue (0.4% solution). 10 µL of the Trypan Blue cell suspension was loaded onto Countess Chamber. Trypan blue membrane permeability was assessed using Countess automated cell counter (Life technologies) using the manufacturer’s protocol.

### Growth Assay

Equal number of cells (1 million cells) were plated in 10 cm dish containing complete media (DMEM) and grown for 9–14 days (as indicated before). After cells have settled down (post 8–10 h), media was removed to get rid of floating dead cells. 10–12 ml of DMEM is added to the 10 cm culture dish and cell growth is monitored using inverted light microscope till 100% confluency. Fitting of sigmoidal curves was performed with an equation of $y\left(t\right)=\frac{a}{1+e^{-k\left(t-t_{0}\right)}}$ , where y(*t*) denotes the cell culture time *t*, a and *k* are fitting parameters, and *t*_0_ defines a lag time of *t*_L_ as *t*_L_ = *t*_0_ −2/*k*. (Nielsen et al., 2001) The error range for the fitting was estimated at the 95% confidence level.

### DNP NMR Spectroscopy

Rotors were transferred in liquid nitrogen directly into the NMR probe that was pre-equilibrated at 100 K. All dynamic nuclear polarization magic angle spinning nuclear magnetic resonance (DNP MAS NMR) experiments were performed on a 600 MHz Bruker Ascend DNP NMR spectrometer/7.2 T Cryogen-free gyrotron magnet (Bruker), equipped with a ^1^H, ^13^C, ^15^N triple-resonance, 3.2 mm low temperature (LT) DNP MAS NMR Bruker probe (600 MHz). The sample temperature was 104 K and the MAS frequency was 12 kHz. The DNP enhancement for the instrumentation set-up for a standard sample of 1.5 mg of uniformly ^13^C, ^15^N labeled proline (Isotech) suspended in 25 mg of 60:30:10 *d*_*8*_-glycerol:D_2_O:H_2_O containing 10 mM AMUPol was between 130 and 140 and a *T*_*B,on*_ of 4.6 s. For ^13^C cross-polarization (CP) MAS experiments, the ^13^C radio frequency (RF) amplitude was linearly swept from 75 to 37.5 kHz with an average of 56.25 kHz. ^1^H RF amplitude was 68–72 kHz for CP, 83 kHz for 90 degree pulse, and 85 kHz for ^1^H TPPM decoupling with phase alternation of ± 15° during acquisition of ^13^C signal. The DNP enhancements were determined by comparing 1D ^13^C CP spectra collected with and without microwaves irradiation. For *T*_B,on_ measurements, recycle delays ranged from 0.1 to 300 s. To determine the *T*_B,on_, the dependence of the recycle delay using saturation recovery on both ^13^C peak intensity or volume was fit to the mono-exponential equation $I_{t}=I_{0}\left(1-e^{\frac{-t}{T_{B,on}}}\right)$ and the stretched-exponential equation $I_{t}=I_{0}\times \left[1-e^{-{\left(\frac{t}{T_{B,on}}\right)}^{\beta }}\right]$ , respectively.

^13^C–^13^C 2D correlations were measured using 20 ms DARR mixing with the ^1^H amplitude at the MAS frequency. A total of 280 complex *t*_1_ points with increment of 25 μs were recorded. For ^13^C-^15^N 1D and 2D correlations, a 24 rotor periods (2 ms) TEDOR sequence was applied with ^13^C and ^15^N pulse trains at 55.5 and 41.7 kHz, respectively. A total of 64 complex *t*_1_ points with an increment of 80 μs were recorded. The recycle delay was 3.9 s and the same ^1^H decoupling was applied. The experimental time required to collect a 2D TEDOR spectra with 32 scans was 2 h and to collect a 2D DARR of 16 scans was 5 h.

### DNP NMR Data Analysis

For 1D experiments, the data were processed using NMRPipe (Delaglio et al., 1995). The real part of the processed spectrum was exported using pipe2txt.tcl command. Peaks were integrated, and the time constants were obtained by least-squares fitting with a single-exponential function. DNP enhancements were determined by peak intensity. For 2D experiments, the TEDOR and DARR data were both apodized with a Lorenz-to-Gauss window function with IEN of 15 Hz and GB of 75 Hz in the *t*_1_ and *t*_2_ time domains. The noise level and peak height from the 2D NMR spectrum was detected by the NMRDraw software for S/N estimation.

## Results and Discussion

### HEK293 Cells Cryopreserved With 10% DMSO Remain Viable During DNP MAS NMR

#### The polarization agent, AMUPol, is not toxic to HEK293 cells in the presence of 10% DMSO

To determine whether AMUPol in the presence of the cryoprotectant 10% *d*_*6*_-DMSO compromised cellular viability, we used a trypan blue dye exclusion test to determine the percentage of cells with intact membranes present in a sample. HEK293 viability was not compromised by replacement of media components with PBS, per-deuteration and addition 10% *d*_6_-DMSO (Supplementary Figure S1A). Moreover, HEK293 viability was not compromised by addition of the polarization agent AMUPol at concentrations up to 50 mM (Supplementary Figure S1B).

##### Cells Cryopreserved With 10% DMSO Retain High Viability After DNP MAS NMR

**FIGURE 1:**
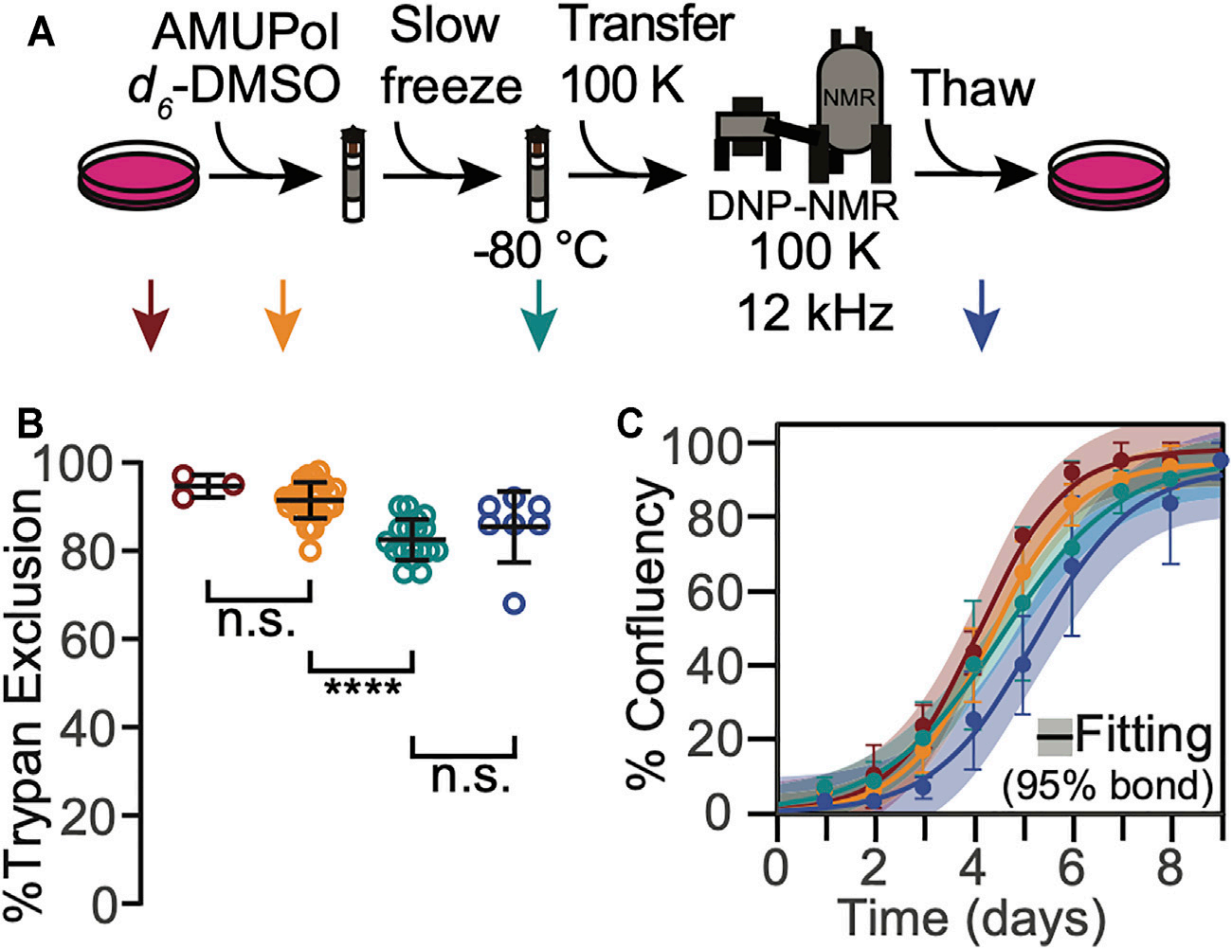
HEK293 cells that are cryopreserved with 10% DMSO are viable throughout the DNP NMR process. **(A)**. Experimental scheme of the DNP NMR sample preparation procedure. Colored arrows indicate points at which sample viability was assessed. Viability was assessed for cells after trypsinization and washing (dark red), after suspension in AMUPol and cryoprotectants (orange), after being frozen at 1 °C per min (green), and after the entire DNP MAS NMR experiment (blue). **(B)** Percentage of cells with trypan impermeable membranes at each sample assessment point, colored as in A. Each point represents an independent sample. Black bars indicate average and standard deviation. Brackets indicate results of two-tailed homoscedastic student’s *t*-tests. (n.s. *p* > 0.05, ****p < 0.0001). **(C)** Growth kinetics as assessed by confluency, colored as in A. The averages and standard deviations of three independent experiments are indicated by circles and error bars, respectively. The best fit of sigmoid is indicated in solid lines and the 95% confidence interval by the shaded area.

To determine whether any of the manipulations required for DNP MAS NMR sample preparation compromise cellular viability when 10% DMSO is used as a cryoprotectant, we assessed trypan blue membrane permeability at several steps of our sample preparation workflow (Figure 1A, arrows). After harvesting adherent cells from tissue culture plates, the cells were rinsed with PBS and pelleted. At this point, cellular membrane integrity as assessed by trypan blue dye exclusion tests was high (95 ± 3%, Figure 1, dark red). Addition of 10% DMSO and AMUPol followed by transfer into 3.2 mm NMR rotors did not significantly decrease membrane integrity (91 ± 4%, Figure 1, orange; *p* = 0.19). Freezing cryoprotected cells at the controlled rate of 1 °C/min slightly compromised membrane integrity as assessed by trypan blue dye exclusion test (decrease of 10 ± 6% to 82 ± 5%, Figure 1B, green, *p* = 1e-5). Post-NMR, trypan blue membrane integrity was indistinguishable from that of slow frozen samples (83 ± 10%, Figure 1, blue, *p* = 1). Cryopreservation of cells with 10% DMSO is therefore compatible with high membrane integrity post-DNP NMR MAS.

The membrane integrity throughout the DNP MAS NMR sample preparation protocol for cells cryoprotected with 10% DMSO was similar, though not identical, to that for cells cryoprotected with 15% glycerol (Ghosh et al., 2021). The membrane integrity of frozen cells was lower than that of fresh cells for cells cryoprotected with either 10% DMSO or 15% glycerol, however the decrease in membrane integrity occurred at different points in the sample preparation. The membrane integrity of cells cryoprotected with 10% DMSO did not decrease upon addition of the cryoprotectant and slightly decreased (10 ± 6%) upon freezing. In contrast, the membrane integrity of cells cryoprotected with 15% glycerol slightly decreased upon addition of the cryoprotectant (5 ± 10%) and was unchanged by freezing. This difference likely reflects the difference in the mechanisms of interaction of the cryoprotectants with cellular membranes. In both cases, the viability of cryoprotected frozen sample, the state that is most representative of the state of the sample during NMR data collection, was the same. Interestingly, the membrane integrity of these sample after DNP MAS NMR was different. It was higher by 14 ± 14% for cells cryopreserved with 10% DMSO than it was for cells cryopreserved with 15% glycerol (*p* = 0.03). However, the loss in membrane integrity after DNP MAS NMR experimentation for cells that were cryopreserved with 15% glycerol is a result of the manipulations required to remove the cells from the rotor, and not the DNP MAS NMR experiment itself. (Ghosh et al., 2021) This indicated that membranes of cells cryopreserved with 10% DMSO were less sensitive to the manipulations required to unpack the rotor than cells cryopreserved with 15% glycerol. This may reflect differences in intracellular ice content, which can recrystallize under slow thawing conditions and damage cells, and/or in changes in diffusion and osmosis across the cellular membrane, which may result in membrane rupture if they exceed the tolerance of the cellular membrane (Pegg, 2007). Overall, this indicates cellular membrane integrity is maintained for cells cryopreserved with 10% DMSO before, during and after DNP MAS NMR. The maintenance of cellular membrane integrity for cells cryopreserved with 10% DMSO and 15% glycerol is similar before and during DNP MAS NMR experimentation and is better for cells that are cryopreserved with 10% DMSO than for cells cryopreserved with 15% glycerol after the DNP MAS NMR experiment.

To determine whether any of the manipulations required for DNP MAS NMR sample preparation compromised cellular propagative ability, we next assessed cellular growth kinetics at each step in our workflow. We found none of the manipulations significantly altered growth kinetics for cells that have been cryoprotected with 10% DMSO (Figure 1C). Cell growth curves were well-fit by a sigmoidal function with lag phase (*R* ^*2*^ = 0.99 ± 0.01). The lag phases and cell growth rates were indistinguishable across all the tested conditions (*p* > 0.26) and all plates reached 100% confluency. Similar to the membrane integrity results, the growth kinetic results for cells cryoprotected with 10% DMSO were similar, though not identical, to those for cells cryoprotected with 15% glycerol (Ghosh et al., 2021). The most notable difference was that exposure to glycerol prolongs the lag phase by 1.5 ± 0.5 days (Ghosh et al., 2021) while exposure to 10% DMSO does not. Otherwise, as for cells cryopreserved with 10% DMSO, no other perturbations significantly altered the growth kinetics. The maintenance of cellular propagative ability for cells cryopreserved with 10% DMSO and 15% glycerol is similar throughout DNP MAS NMR experimentation, although cells cryopreserved with 10% DMSO do not experience a lag phase. This indicates that while both 10% DMSO and 15% glycerol are effective cryoprotectants, 10% DMSO may be a better choice of cryoprotectants for experiments that will benefit from post-NMR cellular growth-based phenotyping.

### Addition of AMUPol to HEK293 Cells Results in DNP Enhancement of all Biomass Components

#### Cells cryopreserved with 10% DMSO and 15% glycerol have similar DNP performance

**FIGURE 2:**
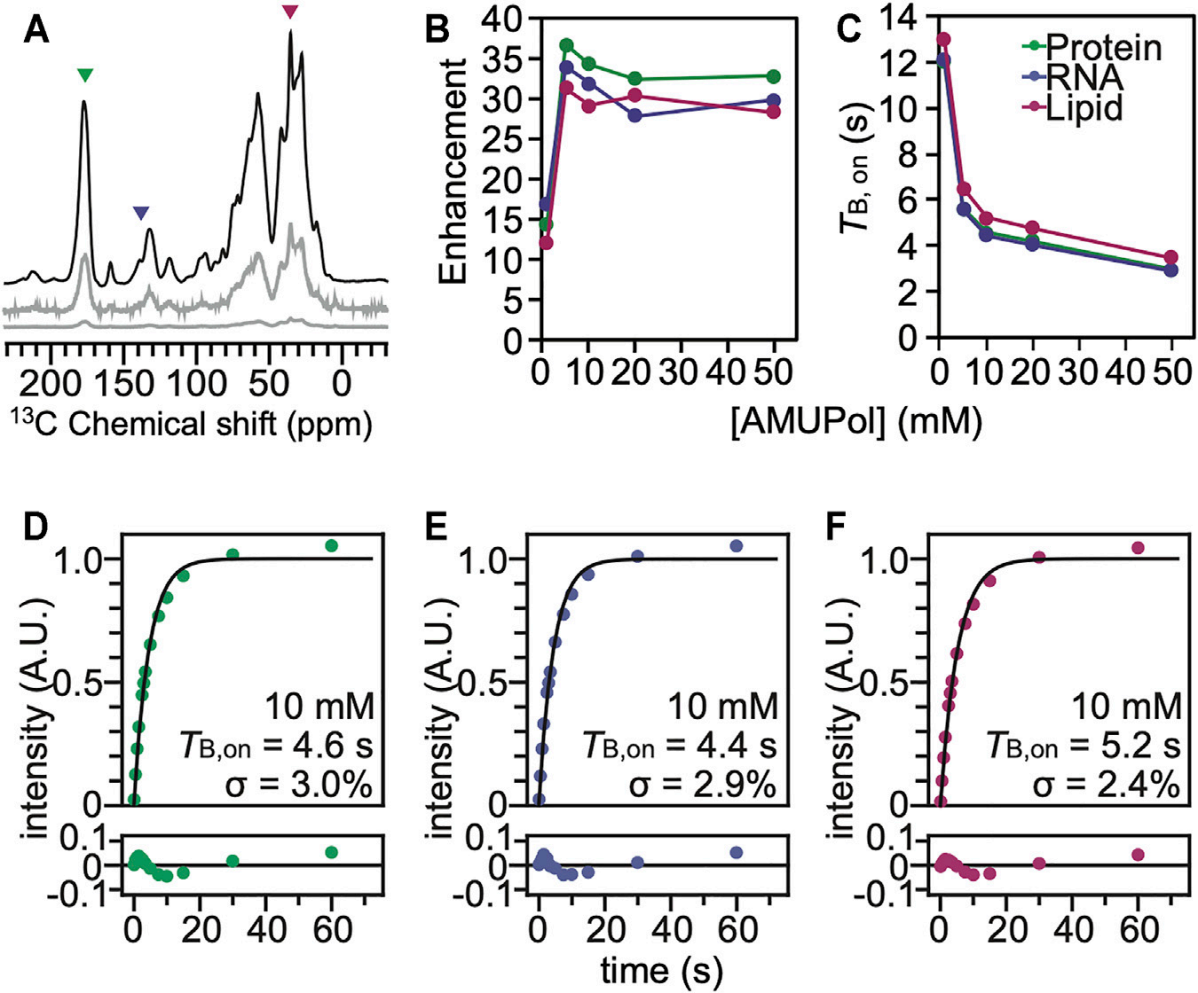
The polarization agent, AMUPol, effectively polarizes all the components of HEK293 cells cryoprotected with 10% DMSO. **(A)** ^13^C cross-polarization spectra of cryopreserved HEK293 cells grown on isotopically enriched media with 10 mM AMUPol at 100 K taken at 600 MHz with 12 kHz magic angle spinning and a recycle delay of 10 s. Displayed spectra are taken with (black) and without (grey) microwave irradiation. The microwave off spectrum is plotted on the same scale as the microwave on spectrum (bottom) and with the intensity multiplied by 10 (middle). Colored arrowheads indicate peaks that are representative of proteins (green), nucleotides (blue) and lipids (pink). **(B)** DNP enhancement and **(C)** *T*_B,on_ values from saturation recovery experiments are dependent upon the AMUPol concentration. Fits of the *T*_B,on_ data to a mono-exponential equation (black line) for different biomass components for cells incubated with 10 mM AMUPol with 10% DMSO as a cryoprotectant. **(D)** The protein component had a *T*_B,on_ value of 4.6 s with a regression error (lower plot) of 3.0%. **(E)** The nucleotide component had a *T*_B,on_ value of 4.4 s with a regression error (lower plot) of 2.9%. **(F)** The lipid component had a *T*_B,on_ value of 5.2 s with a regression error (lower plot) of 2.4%.

Using characteristic peaks in the NMR spectra as reporters of the different cellular biomass components (Ghosh et al., 2021), we assessed DNP performance for cells that had been incubated with AMUPol and cryopreserved using 10% DMSO. We collected ^13^C cross-polarization (CP) spectra with and without microwave irradiation to determine the DNP enhancements for HEK293 cells incubated with a range of AMUPol concentrations. We determined DNP enhancements for peaks in the ^13^C CP spectra that are representative of the major biomass components of HEK293 cells; proteins, nucleotides and lipids (Figure 2). While some chemical moieties, like carbonyls, are found in more than one major biomass component—the contribution of lipid head groups could be up to 7% of the “protein” peak and the contribution of aliphatic side chains could be up to 22% of the “lipid” peak—these peaks serve as quantitative proxies for the different biomass components (Ghosh et al., 2021). We found that the DNP enhancements for intact HEK293 cells that were cryopreserved with 10% DMSO reached a maximum value of 39 for the protein component and addition of 5 mM AMUPol sufficed to attain this enhancement. Addition of higher concentrations of AMUPol to the sample did not significantly alter the DNP enhancement across all biomass components (Figure 2B). The DNP enhancements for cells cryoprotected with 10% DMSO and then incubated with AMUPol are very similar to those for cells cryoprotected with 15% glycerol and then incubated with AMUPol (Ghosh et al., 2021). The dependence of the DNP enhancements on the concentration of AMUPol for cells cryoprotected with 10% DMSO and 15% glycerol were indistinguishable (*p* = 0.44, *n* = 5). We next assessed the DNP build-up times (*T*_B,on_) (Pinon et al., 2017) for cells that had been incubated with AMUPol and cryopreserved using 10% DMSO. As expected, we found that the value of *T*_B,on_ decreased with increasing AMUPol concentrations. The *T*_B,on_ for the protein component of cells incubated with 5 mM AMUPol was 5.6 s and decreased to 3.0 s for cells that were incubated with 50 mM AMUPol. The dependence of *T*_B,on_ values on the concentration of AMUPol for cells cryoprotected with 10% DMSO are very similar to those for cells cryoprotected with 15% glycerol. The dependence of *T*_B,on_ values on concentration of AMUPol for cells cryoprotected with 10% DMSO and 15% glycerol were indistinguishable (*p* = 0.16, *n* = 5). Interestingly, the maximal enhancement for proteins inside intact cells, regardless of the cryoprotectant, is ∼40 which is half of the maximal enhancement for proteins in cellular lysates, where the plasma membrane of the cell doesn’t present an accessibility barrier. The higher maximal enhancements and the much steeper dependance of *T*_B,on_ on AMUPol concentration for cellular lysates than for intact cells that were incubated with AMUPol suggests that, as was previously observed for cells cryoprotected with 15% glycerol, the AMUPol concentration inside of cells cryoprotected with 10% DMSO is lower than the concentration of AMUPol that was added to the sample. This indicates that AMUPol is heterogeneously distributed in samples of intact cells cryopreserved with 10% DMSO.

### AMUPol is Heterogeneously Distributed in Cells Cryopreserved in Both 10% DMSO and 15% Glycerol

To assess the homogeneity of the AMUPol concentration throughout each biomass component, we used the regression error of the fit of the *T*_B,on_ data to a mono-exponential equation (Ghosh et al., 2021) as well as a stretched exponential function where β describes the degree of deviation from an exponential fit (Pinon et al., 2017; Rankin et al., 2019). The regression error is a modestly more sensitive measure for deviation from a monoexponential and the regression error and the β factor are strongly anti-correlated. Both the regression error and β are reported in Supplementary Table S2. If the concentration distribution of AMUPol is heterogenous, there will be a mixture of underlying *T*_B,on_ values which will increase the regression error. For reference, the regression error of the fit of the *T*_B,on_ data to a mono-exponential function of the amino acid proline suspended in a matrix of 60:30:10 (v/v) glycerol:D_2_O:H_2_O with 10 mM AMUPol was 0.5% and represents the error expected from experimental noise (Ghosh et al., 2021). For intact cells cryopreserved with 10% DMSO, the regression error for protein was 2.8 ± 0.8% and for nucleotide the regression error was 2.6 ± 0.6% (indistinguishable from protein, *p* = 0.19, *n* = 5), while the regression error for lipid was 2.2 ± 0.6% (lower than protein and nucleotide, *p* < 0.003, *n* = 5) (Supplementary Table S2). These regression errors were indistinguishable from those for intact cells cryopreserved with 15% glycerol (*p* = 0.74) and were significantly larger than the regression error for lysed cells, where the plasma membrane does not present a barrier to accessibility (*p* = 0.005) as well as for intact cells where AMUPol was introduced inside the cell by electroporation (*p* = 0.004) across all biomass components. When AMUPol is dispersed homogenously throughout the sample, the regression errors are small. The larger regression errors for cell incubated with AMUPol and cryopreserved with 10% DMSO indicates the concentration distribution of AMUPol is more heterogenous in these samples than in samples of lysed cells or cells where AMUPol is delivered by electroporation. DMSO is sometimes used to improve cellular permeability of small molecules. If DMSO improves delivery of AMUPol to cells, the regression error for cells incubated with AMUPol and cryopreserved with 10% DMSO should be smaller than those for cells incubated with AMUPol and cryopreserved with 15% glycerol. However, the regression errors are indistinguishable. This indicates that the choice of cryoprotectant does not alter the delivery of the polarization agent to the cell. Finally, it is possible that inhomogeneities in the dispersion of the radical could result from the formation of ice crystals, rather than from larger scale inhomogeneities that result from physical exclusion of the radical from cell interiors by the plasma membrane. However, this is unlikely. Ice crystal formation inside cells kills cells and these samples had high post thaw viability. Moreover, when AMUPol is introduced into cells by electroporation, which circumvents the physical exclusion of the radical from the cell interior, cryoprotected cells also have high post-thaw viabilities, along with homogenous radical dispersions and high DNP enhancements (Ghosh et al., 2021). Thus, incubation of cells with AMUPol results in inhomogenous distribution of the AMUPol throughout the sample, regardless of the choice of cryoprotectant.

**FIGURE 3:**
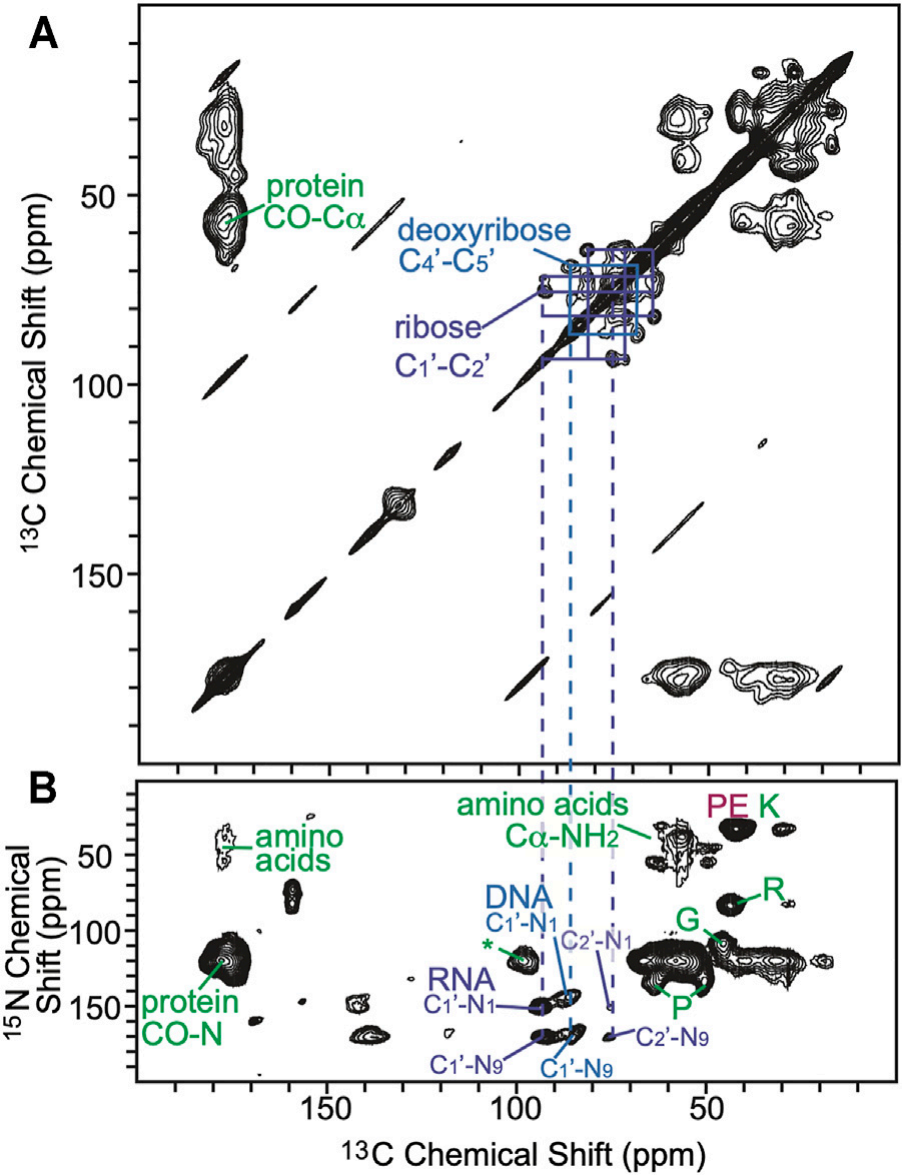
**(A)** 2D homonuclear correlation spectra (DARR) of cells cryoprotected with DMSO. Selected ^13^C–^13^C correlations from carbons in the ribose (purple) and deoxyribose (blue) rings of RNA and DNA are annotated. **(B)** 2D heteronuclear correlation spectra (zTEDOR) of cells cryoprotected with DMSO. Selected ^13^C-^15^N correlations from the protein back bone and sidechains (green), from RNA (purple), from DNA (blue) and from lipid (pink) are annotated. The signal to noise ratios of selected peaks are reported in Supplementary Table S1.

To further explore the distribution of AMUPol in samples of intact cells cryopreserved with 10% DMSO, we collected DNP-enhanced 2D ^13^C-^15^N correlation spectra (TEDOR) (Jaroniec et al., 2002) and assessed the signal to noise ratios for biomass components with different cellular distributions. We compared the normalized peak intensities of cytosolic and nucleic components for cells incubated with AMUPol and cryopreserved with DMSO to those of lysed samples and intact samples that were either electroporated or incubated with AMUPol and cryopreserved with 15% glycerol (Ghosh et al., 2021). The TEDOR peak intensities were normalized to either the ribose-purine peak of RNA or DNA for each sample to control for differences in DNP-enhancements and cross-polarization efficiencies. TEDOR spectra have distinct peaks for DNA, RNA, protein backbone sites, protein side chain moieties, and free amino acids (Figure 3). DNA is located only in the nucleus, while RNA, proteins and free amino acids are entirely or largely cytoplasmically localized (i.e. more than 80% of the protein content of a cell is non-nuclearly localized). (Shaiken and Opekun, 2014) In addition to the RNA and DNA ribose purine and pyrimidine peaks, we determined peak intensities for the amide-carbonyl and amide-C_α_ sites for both proteins and free amino acids as well as the carbon-nitrogen bonds in the protein side chains of arginine and glycine (Supplementary Table S1). When the intensity of the amino acids peaks are compared to the ribose-purine peak of RNA, the ratio of the cross-peak intensities for the sample cryopreserved with DMSO were similar to those for the cellular lysate and intact cellular samples that had been prepared with 15% glycerol as the cryoprotectant, regardless of the method of AMUPol delivery (incubation, electroporation or addition to lysed cells) (*p* > 0.06, student’s paired *t*-test) (Supplementary Table S1) (Ghosh et al., 2021). As an example, the glycine C_α_-N cross peak was 1.23, 1.17, 1.43, and 1.16 times more intense than the ribose-purine cross peak of RNA for lysed cell, intact cells electroporated with AMUPol, intact cells incubated with AMUPol in 15% glycerol, and intact cells incubated AMUPol in DMSO, respectively. The similarity of the relative cross-peak intensities for the cytoplasmically-located biomass components across different approaches to sample preparation indicated that the cytoplasmic distribution of AMUPol is similar for all these samples. When the intensity of the amino acid and RNA peaks are compared to the deoxyribose-purine peak of DNA, we found that the sample incubated with AMUPol and cryopreserved with DMSO had indistinguishable peak intensity ratios to those for the sample incubated with AMUPol and cryopreserved with 15% glycerol (*p* = 0.24, Student’s t-test, paired) and very different peak intensity ratios from the lysed and electroporated cells (*p* < 0.02, Student’s t-test, paired). For example, the glycine C_α_-N cross peak is 1.75, 1.80, 2.55 and 2.67 times more intense than the deoxyribose-purine cross peak of DNA for lysed cell, intact cells electroporated with AMUPol, intact cells incubated with AMUPol in 15% glycerol and intact cells incubated AMUPol in DMSO, respectively. The DNA peaks for intact cells incubated with AMUPol and then cryoprotected with either DMSO or glycerol were less intense than expected; the ratios of peak intensities for cytoplasmic to nuclear components were larger by 53% ± 19% (*p* < 0.05) (Supplementary Table S1 and reference 14). Because the nuclear envelope is known to be permeable to AMUPol in intact cells (Ghosh et al., 2021), this suggested that the AMUPol concentration in the nucleus of cells incubated with AMUPol, regardless of choice of cryoprotectant, is lower than the concentration of AMUPol in the cytoplasm. Overall, AMUPol is heterogeneously distribution when intact cells are incubated with AMUPol. While AMUPol can polarize all the biomass components, including DNA, the relatively lower intensity of the DNA peaks combined with larger regression errors indicate that there is an AMUPol concentration gradient inside these cells. Although DMSO can improve delivery of small molecules to cells, there is no indication that DMSO improves delivery of AMUPol. The choice of cryoprotectant does not alter the delivery of the polarization agent to the cell.

## Conclusion

Prior work established that sample conditions that favor efficient DNP enhancements are compatible with cellular viability and that the magnitude of the sensitivity enhancements is high enough to enable detection of a protein at micromolar concentrations in experimentally tractable experimental times. However, in that work, 15% glycerol, rather than the more commonly used 10% DMSO, was used as the cellular cryoprotectant. Moreover, incubation of cells cryoprotected 15% glycerol with AMUPol resulted in an inhomogeneous distribution of the polarization agent, AMUPol, through the cellular biomass, which will result in a spatial bias of the NMR peak intensities. Because 10% DMSO is not only the most used cryoprotectant for mammalian cells, but also because DMSO is often used to improve delivery of molecules to cells, we sought to characterize the DNP performance of cells that were incubated with AMUPol and cryoprotected with 10% DMSO. We found that, like cells preserved with 15% glycerol, cells preserved with 10% DMSO retain high viability during DNP MAS NMR experiments. Moreover, cells preserved with 10% DMSO were less sensitive to the manipulations required to unpack cells from the NMR rotor, suggesting that it may be a better cryoprotectant for experiments that require post-NMR growth-based phenotyping. However, DMSO did not improve the dispersion of AMUPol throughout the cellular biomass. Cells preserved with 15% glycerol and with 10% DMSO had similar DNP performance for both the maximal DNP enhancements as well as the inhomogeneous dispersion of AMUPol throughout the cellular biomass. Therefore, we establish that 10% DMSO and 15% glycerol can be used interchangeably for DNP-assisted MAS NMR of intact viable mammalian cells.

Here we examined the cryopreservation and DNP properties for cells that were cryopreserved using concentrations of cryoprotectants at their established working concentrations of 10% for DMSO and 15% glycerol. At these working concentrations, the cryoprotective properties and DNP performance were indistinguishable. However, addition of different percentages of the same cryoprotectants can dramatically affect viability. Prior work established that suspension of cells in 60% glycerol, a percentage commonly used in biological DNP samples, or 60% DMSO both resulted in significant losses of membrane integrity and a complete loss of propagative ability (Ghosh et al., 2021). Thus, both cryoprotectants are compatible with high DNP enhancements on intact viable mammalian cells, but only at concentrations appropriate for cellular cryoprotection.

Because DMSO and glycerol have indistinguishable cryoprotective properties and DNP performance, both cryoprotectant systems are well-suited for in cell DNP MAS NMR of mammalian cells. Because both cryoprotectants fulfill the major requirements of viability maintenance and DNP efficiency, the choice of cryoprotectant depends upon question under investigation. For example, long term exposure to even low concentrations of DMSO is toxic. Although this is unlikely to be a major concern since the exposure to high concentrations of DMSO is transient, glycerol does not have the same toxicity profile and may be a more appropriate choice for sensitive cellular systems. However, we observed that cells preserved with 10% DMSO were less sensitive to the manipulations required to unpack cells from the NMR rotor, suggesting that DMSO may be a better cryoprotectant for experiments that require post-NMR growth-based phenotyping. Interestingly, DMSO and glycerol interact differently with the cell membrane. In general, DMSO de-solvates lipid membranes, increases the chain melting temperature (Yu and Quinn, 1995), induces water pores and increases floppiness in lipid membranes (Notman et al., 2006) while glycerol affects lipid hydration only to the same degree as it does of bulk water (Schrader et al., 2016) and does not alter the chain melting temperature of lipid membranes (McDaniel et al., 1983). Thus, while macroscopically DMSO may protect cellular membranes from rupturing, microscopically, glycerol may better preserve the local character of the membrane which could be particularly important for investigations of membrane-associated biomolecules and may be a more appropriate cryoprotectant for questions that require maintenance of the local structural integrity of lipid membranes. Finally, the work presented here was done on cells that were grown on isotopically enriched media. Therefore, the spectral contribution of the cryoprotectants to the signal were negligible because the ^13^C content from natural abundance carbon in the cryoprotectants accounts for ∼0.1% of the volume of the sample. However, for samples where the target molecule is at concentrations that are low enough that signals from natural abundance components make a significant contribution to the spectra (Costello et al., 2019), the contribution of the cryoprotectant peak to the spectra becomes a consideration. DMSO has one ^13^C peak at 40 ppm and glycerol has two ^13^C peaks at 65 and 75 ppm (Supplementary Figure S2). DMSO overlaps with protein sidechains while glycerol overlaps with the alpha carbons of some amino acids and the ribose ring of nucleic acids. While there is currently no source for ^13^C-depleted *d*_6_-DMSO, ^13^C-depleted *d*_8_-glycerol is commercially available and reduces the ^13^C content of the cryoprotectant by an order of magnitude, which may make glycerol a more attractive choice for sensitivity-limited samples. Because both DMSO and glycerol are both well-suited for in-cell DNP MAS NMR, the choice of cryoprotectant system can be tailored to the system under investigation.

Finally, although DMSO is often used to improve delivery of molecules to cells, it did not improve the delivery of the polarization agent, AMUPol, to cells. The DNP enhancements, *T*_B,on_ values and residual errors for samples preserved with 10% DMSO were indistinguishable from those for sample preserved with 15% glycerol. We considered the possibility that the delivery of AMUPol was improved in the presence of DMSO, but the reductive environment of the cell inactivated the AMUPol inside the cell (Jagtap et al., 2015; Karthikeyan et al., 2018), resulting in similar DNP performance. For cells that were cryopreserved with 15% glycerol, the DNP performance for cells incubated with AMUPol was relatively constant for room temperature incubation times of up to 2 h because the plasma membrane is semi-permeable to AMUPol and as the small amount that enters the cell is reduced, it is replenished by the large concentration of AMUPol in the interstitial space (Ghosh et al., 2021). More generally, the reduction of AMUPol by mammalian cells is slow relative to the sample preparation time; the half-life of AMUPol in intact cells is about an hour (Ghosh et al., 2021). Additionally, the *T*_B,on_ values for samples prepared with both 10% DMSO and 15% glycerol are indistinguishable. Because the bi-nitroxide radicals in AMUPol, are reduced independent of each other. The monoradical form of AMUPol is DNP-silent, but still contributes to paramagnetic relaxation (McCoy et al., 2019). The accumulation of monoradical forms of AMUPol explains the observation that maximal enhancement for intact mammalian cells, where the AMUPol is reduced during the sample preparation time, is about half of the maximal enhancement for lysed cells, which can be flash frozen which prevents the build-up of monoradical forms, yet have similar *T*_B,on_ values (Ghosh et al., 2021). The monoradical forms of AMUPol shorten the *T*_B,on_ without contributing to the enhancement. Thus, if more AMUPol is delivered to cells in the 10% DMSO condition but then is also reduced by cells, the enhancements could be similar but the *T*_B,on_ values for those preparations should be shorter. However, this is not the case. Therefore, the presence of 10% DMSO did not improve delivery of a polarzation agent AMUPol to HEK293 cell.

Because the sensitivity enhancements from DNP rely upon proximity to a polarization agent, DNP-enhanced MAS NMR experiments are biased towards observation of molecules that are accessible to polarization agents. Here we found that for cells incubated with AMUPol and cryoprotected with 10% DMSO, a minority of the AMUPol enters the cell; the peak intensities for DNA are lower than expected and the *T*_B,on_ fits indicate that the AMUPol concentration is heterogenous. Thus, while the identity, stoichiometry, concentrations and organization of the cellular components for cells incubated with AMUPol are all maintained, the experimental read-out from such samples are spatially biased, just like they are for cells incubated with AMUPol and cryoprotected with 15% glycerol. Data from experiments performed on intact cells incubated with AMUPol are qualitative rather than quantitative. While any observed conformation inside cells incubated with AMUPol exists, the relative populations of different conformations cannot be inferred from peak intensities. For in cell work where such quantitative information is required, alternative approaches that result in homogenous dispersion of the polarization agent—like electroporation—are more appropriate (Ghosh et al., 2021). Investigation of protein conformations inside viable cells using DNP MAS NMR creates an experimental system with the ability to tightly couple genotypes, phenotypes and environments (e.g., presence/absence of a drug) to specific structures or structural ensembles. Cryoprotection of cells using the commonly used cryoprotectant, DMSO, is compatible with in cell DNP MAS NMR.

## Data Availability

The original contributions presented in the study are included in the article/Supplementary Material, further inquiries can be directed to the corresponding author.
