# Supplementary figures and images for: In-Cell NMR of Intact Mammalian Cells Preserved with the Cryoprotectants DMSO and Glycerol Have Similar DNP Performance
Source: Front Mol Biosci. 2022 Jan 25;8:789478. doi: 10.3389/fmolb.2021.789478 (PMC8824258; doi:10.3389/fmolb.2021.789478)

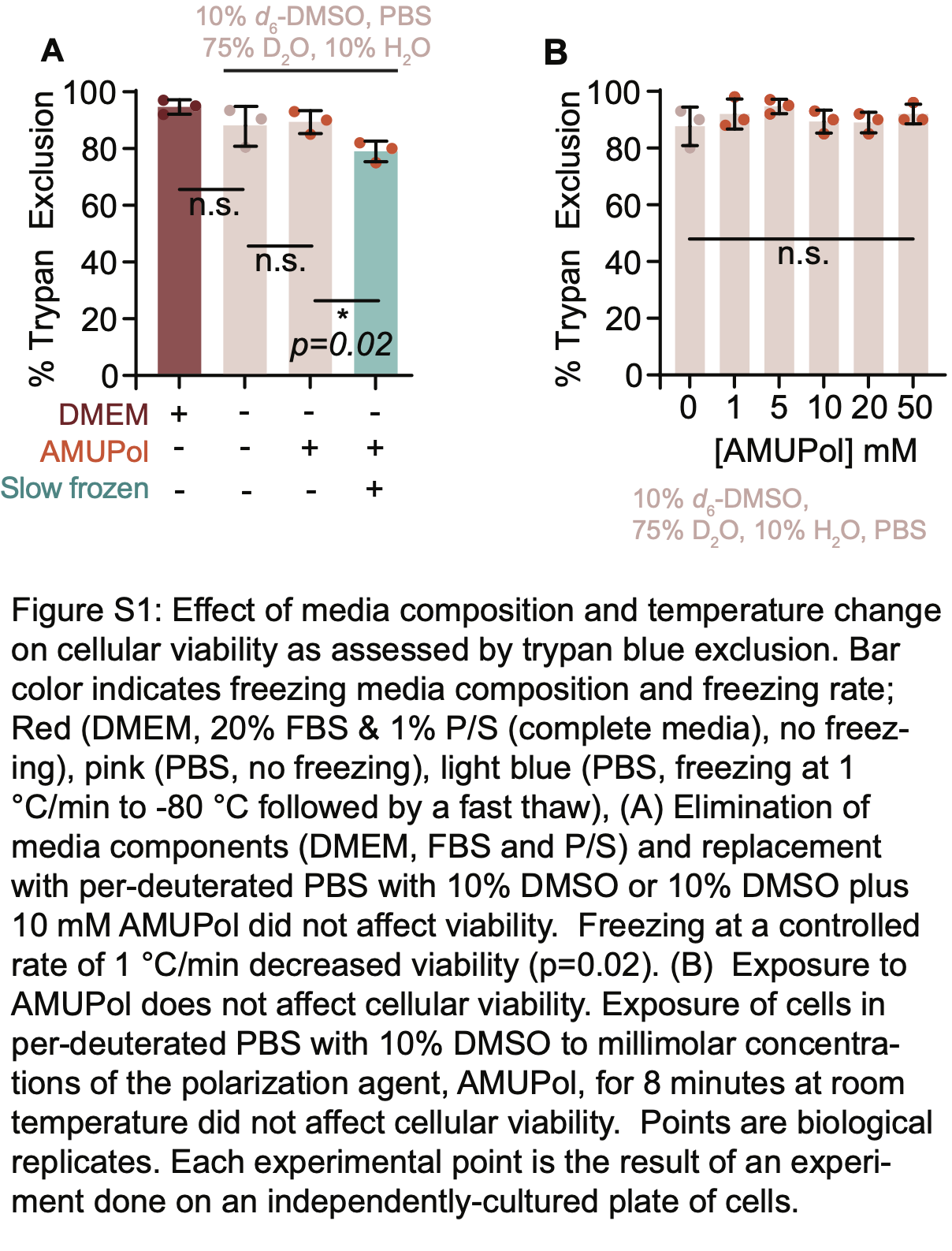

Supplement: Supplementary file 1 [file Image1.TIFF]

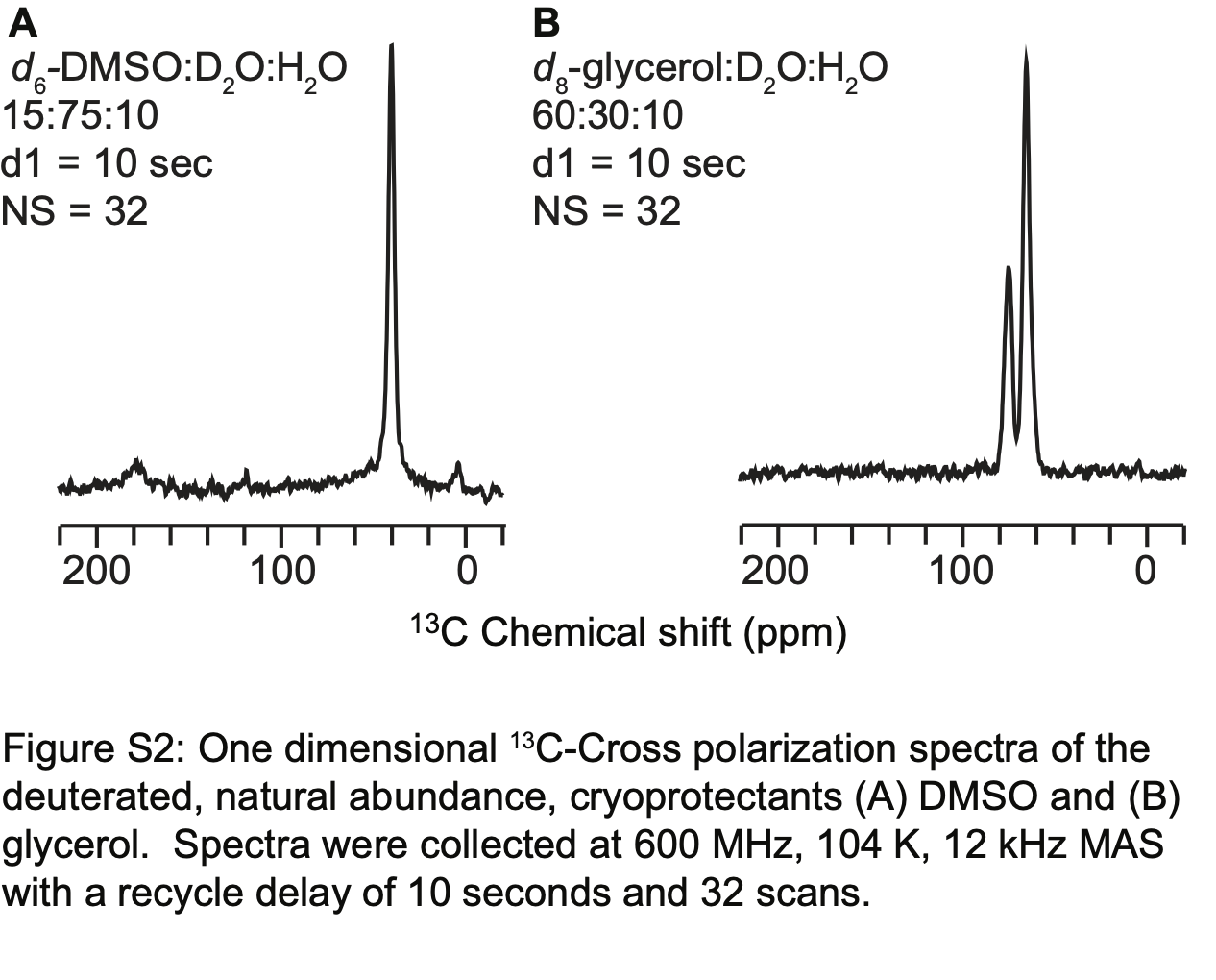

Supplement: Supplementary file 4 [file Image2.TIFF]
