# Supplementary material for: In-Cell NMR of Intact Mammalian Cells Preserved with the Cryoprotectants DMSO and Glycerol Have Similar DNP Performance
Source: Front Mol Biosci. 2022 Jan 25;8:789478. doi: 10.3389/fmolb.2021.789478 (PMC8824258; doi:10.3389/fmolb.2021.789478)
Supplement: Supplementary file 2 [file Table1.DOCX]

**Table S1: Signal to noise ratios for biomass components in two-dimensional spectra.**

| Samples | Incubation, *ε*  = 48.5 | | | | |
| --- | --- | --- | --- | --- | --- |
| Biomass  components | Peak location | | | SNR | |
|  | *t*_2_ (ppm) | *t*­_1_ (ppm) | | AV | ratio |
| TEDOR | NS = 32 | | | | |
| DNA C_1_'-N_1_ | 86.1 | | 144.0 | 47.4 | 0.4 |
| DNA C_1_'-N_9_ | 85.0 | | 171.1 | 66.2 | 0.6 |
| RNA C_1_'-N_1_ | 93.6 | | 151.0 | 108.9 | 1.0 |
| RNA C_1_'-N_9_ | 92.7 | | 170.9 | 161.0 | 1.5 |
| Amino Acid C_α_-N | 56.1 | | 38.3 | 62.7 | 0.6 |
| Amino Acid CO-N | 177.2 | | 39.5 | 19.5 | 0.2 |
| Protein C_α_-N | 58.0 | | 120.1 | 764.6 | 7.0 |
| Protein CO-N | 177.8 | | 120.0 | 609.0 | 5.6 |
| Arg C_ζ_-N_ξ_ | 43.8 | | 83.5 | 187.9 | 1.7 |
| Gly C_α_-N | 45.4 | | 108.8 | 126.7 | 1.2 |
| DARR | NS = 16 | | | | |
| DNA C_4_'-C_5_' | 86.8 | | 68.6 | 47.3 | 0.4 |

Direct dimension (*t*_2_): ^13^C Chemical shift in ppm for TEDOR and DARR experiments.

Indirect dimension (*t*_1_): ^15^N chemical shift in ppm for TEDOR experiments and ^13^C chemical shift in ppm for DARR experiments.

Enhancement of protein (*ε*): Calculated from the CO peak intensity ratio of ^1^H-^13^C 1D CP experiments (*d*_1_ = 10 s) with and without microwave.

The signal to noise is reported as the absolute value (AV) and as a ratio relative to the SNR of the RNA C1’-N1’ peak to facilitate comparison of the SNR across different sample preparation approaches.

Spectra are referenced to DSS.

* SNR ratios are those of the spectra collected with the indicated number of scans.

Data for all samples prepared with 15% glycerol are presented, in the same format, in the supplemental information of Ghosh, R., Xiao, Y., Kragelj, J. & Frederick, K.K. In-Cell Sensitivity-Enhanced NMR of Intact Viable Mammalian Cells. *Journal of the American Chemical Society* **143**, 18454-18466 (2021) and are available free of charge at: [https://pubs.acs.org/doi/10.1021/jacs.1c06680](https://pubs.acs.org/doi/10.1021/jacs.1c06680?goto=supporting-info).
