# Supplementary material for: In-Cell NMR of Intact Mammalian Cells Preserved with the Cryoprotectants DMSO and Glycerol Have Similar DNP Performance
Source: Front Mol Biosci. 2022 Jan 25;8:789478. doi: 10.3389/fmolb.2021.789478 (PMC8824258; doi:10.3389/fmolb.2021.789478)
Supplement: Supplementary file 3 [file Table2.DOCX]

| AMUPol delivery | Incu  bation time (min) | [AMU  Pol]  mM | protein | | | | | | RNA | | | | | | Lipid | | | | | |
| --- | --- | --- | --- | --- | --- | --- | --- | --- | --- | --- | --- | --- | --- | --- | --- | --- | --- | --- | --- | --- |
|  |  |  | 𝜀 | mono-exp | | stretch-exp | | | 𝜀 | mono-exp | | stretch-exp | | | 𝜀 | mono-exp | | stretch-exp | | |
|  |  |  |  | *T*_B,on_ | reg. | *T*_B,on_ | 𝝱 | reg. |  | *T*_B,on_ | reg. | *T*_B,on_ | 𝝱 | reg. |  | *T*_B,on_ | reg. | *T*_B, on_ | 𝝱 | reg. |
| incubation | 8 | 1 | 14.3 | 12.0 | 1.5% | 13.0 | 0.91 | 0.7% | 16.9 | 12.1 | 1.6% | 12.7 | 0.94 | 1.4% | 12.0 | 13.0 | 1.3% | 13.8 | 0.93 | 0.7% |
| incubation | 8 | 5 | 36.7 | 5.6 | 2.5% | 6.3 | 0.85 | 0.9% | 34.0 | 5.6 | 2.5% | 6.2 | 0.86 | 1.2% | 31.3 | 6.5 | 2.0% | 7.1 | 0.89 | 1.0% |
| incubation | 8 | 10 | 34.3 | 4.6 | 3.0% | 5.2 | 0.82 | 1.0% | 31.9 | 4.4 | 2.9% | 5.0 | 0.83 | 1.1% | 29.1 | 5.2 | 2.4% | 5.7 | 0.86 | 1.1% |
| incubation | 8 | 20 | 32.5 | 4.2 | 3.5% | 4.9 | 0.79 | 1.0% | 27.9 | 4.0 | 3.0% | 4.5 | 0.82 | 1.2% | 30.4 | 4.8 | 2.8% | 5.4 | 0.84 | 1.1% |
| incubation | 8 | 50 | 32.9 | 3.0 | 3.5% | 3.3 | 0.78 | 0.9% | 29.8 | 2.9 | 3.1% | 3.2 | 0.81 | 1.0% | 28.3 | 3.5 | 2.7% | 3.8 | 0.83 | 1.1% |

**Table S2**: Summary of DNP MAS NMR experiments.

Mono-exp reports values determined from a fit to a mono-exponential function. Stretch-exp reports values determined from a fit to a stretched exponential function.

*T*B,on is reported in units of seconds and was determined from 13 time points that ranged from 0.5~60 sec. (0.1, 0.5, 1.0, 1.5, 2.5, 3.0, 3.5, 5.0, 7.5, 10, 15, 30, and 60 sec, respectively). Additional points with longer delays were collected when T*B,on* exceeded 10 seconds.

reg. is the regression error, determined as reported in the methods section.

Data for all samples prepared with 15% glycerol are presented, in the same format, in the supplemental information of Ghosh, R., Xiao, Y., Kragelj, J. & Frederick, K.K. In-Cell Sensitivity-Enhanced NMR of Intact Viable Mammalian Cells. *Journal of the American Chemical Society* **143**, 18454-18466 (2021) and are available free of charge at: [https://pubs.acs.org/doi/10.1021/jacs.1c06680](https://pubs.acs.org/doi/10.1021/jacs.1c06680?goto=supporting-info).
